# Chitosan Poly(vinyl alcohol) Methacrylate Hydrogels for Tissue Engineering Scaffolds

**DOI:** 10.1021/acsabm.3c01209

**Published:** 2024-02-21

**Authors:** Nghia Le Ba Thai, Henry T. Beaman, Megan Perlman, Ernest E. Obeng, Changling Du, Mary Beth B. Monroe

**Affiliations:** † Department of Biomedical and Chemical Engineering, Syracuse Biomaterials Institute, and BioInspired Syracuse: Institute for Material and Living Systems, Syracuse University, Syracuse, New York 13244, United States

**Keywords:** Hydrogels, Tissue Engineering, Chitosan, Poly(vinyl alcohol), Cytocompatbility, Antimicrobial

## Abstract

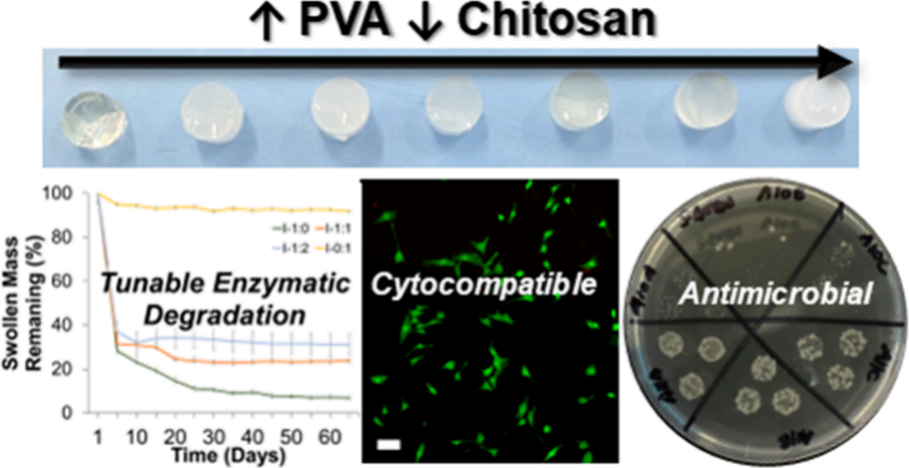

A major challenge in tissue engineering scaffolds is controlling scaffold degradation rates during healing while maintaining mechanical properties to support tissue formation. Hydrogels are three-dimensional matrices that are widely applied as tissue scaffolds based on their unique properties that can mimic the extracellular matrix. In this study, we develop a hybrid natural/synthetic hydrogel platform to tune the properties for tissue engineering scaffold applications. We modified chitosan and poly(vinyl alcohol) (PVA) with photo-cross-linkable methacrylate functional groups and then synthesized a library of chitosan PVA methacrylate hydrogels (ChiPVAMA) with two different photoinitiators, Irgacure 2959 (I2959) and lithium phenyl-2,4,6-trimethylbenzoylphosphinate (LAP). ChiPVAMA hydrogels showed tunability in degradation rates and mechanical properties based on both the polymer content and photoinitiator type. This tunability could enable their application in a range of tissue scaffold applications. In a 2D scratch wound healing assay, all hydrogel samples induced faster wound closure compared to a gauze clinical wound dressing control. NIH/3T3 cells encapsulated in hydrogels showed a high viability (∼92%) over 14 days, demonstrating the capacity of this system as a supportive cell scaffold. In addition, hydrogels containing a higher chitosan content demonstrated a high antibacterial capacity. Overall, ChiPVAMA hydrogels provide a potential tissue engineering scaffold that is tunable, degradable, and suitable for cell growth.

## 1 Introduction

Tissue engineering involves the combination of cells, scaffolds, and bioactive factors to create replacements for tissues or organs damaged by disease or trauma.^1,2^ Despite tremendous efforts to translate tissue engineering approaches over the last two decades, a minimal number of clinical products are available to use due to hurdles in achieving biological functions of tissues along with host biocompatibility.^3,4^ To address these problems, it is critical to strengthen each different aspect of tissue engineering, including controlling stem cell development, enhancing bioactivity by improving delivery mechanisms of bioactive factors, and enriching tissue scaffold properties to better mimic native tissues.^5^

Tissue scaffolds provide a microenvironment to support cells, as they construct new tissue during healing. Thus, the main requirements for scaffolds are biocompatibility and biodegradability. The biodegradation rate should be complementary to the regeneration rate of native tissue while providing adequate mechanical strength to support surrounding tissues. In addition, the scaffolds should have porous structure to allow nutrient transfer and waste removal for cells within the system.^6,7^ One of the key challenges in tissue scaffold development is controlling the degradation rate to match tissue regeneration while maintaining cell viability over time.^8^ Several hydrogel platforms fabricated from natural and synthetic polymers have been developed to overcome this challenge.^9,10^ Hydrogels are three-dimensional matrices extensively applied in tissue engineering scaffolds due to their similarity to tissue extracellular matrix, high permeability for nutrient transfer, and suitability for cell encapsulation. In addition, hydrogels often provide high biocompatibility, degradability, and diversity in structure that is easy to modify.^11,12^

Natural polymers have been used to fabricate hydrogels due to their biocompatibility and biodegradability.^13,14^ They also have better bioactivity compared to synthetic polymers. Synthetic polymers are frequently applied to construct hydrogels because of their hydrophilicity and adjustable mechanical properties.^15,16^ The combination of natural and synthetic polymers could yield flexible hybrid hydrogels with tunable degradation and mechanical properties to provide improved tissue engineering scaffolds.^17,18^

Chitosan is a naturally occurring polysaccharide broadly used in biomedical applications, such as tissue engineering scaffolds,^19^ drug delivery,^20^ and wound healing.^21^ Chitosan-based hydrogels could provide an appropriate environment for cells to develop, and they undergo enzymatic degradation, which could enable cell-responsive scaffold clearance over time.^22−24^ Additionally, the hydroxyl and amine groups in the chemical structure allow for chitosan modification with other materials.^25,26^ While chitosan has many desirable functions for use in healing, including procoagulation and antimicrobial properties,^27−29^ its use suffers from inconsistent batch-to-batch properties and low solubility.^30^ Poly(vinyl alcohol) (PVA) is a water-soluble synthetic polymer that has been frequently applied in tissue engineering and wound healing due to its versatile mechanical properties and biocompatibility.^31−33^ However, PVA does not promote protein adsorption or subsequent cell adhesion, and it is biostable.^34,35^ Here, we aimed to blend modified chitosan and PVA to fabricate hybrid hydrogels and to reduce these limitations.

To enable facile fabrication, these hydrogels were synthesized by using photopolymerization methods. Photopolymerization is widely used in biomedical applications because the reactions can occur at room temperature within short timeframes.^36,37^ This process produces minimal heat, which allows for direct encapsulation of cells within the interior of scaffolds during polymerization with improved cell viability and reduced protein digestion.^38−40^ Briefly, upon exposure to light, the photoinitiator absorbs light and converts this energy to chemical energy to form free radicals. These reactive intermediates react with vinyl bonds in the solution to initiate the cross-linking process.^41^ In this study, two kinds of photoinitiators were used during photopolymerization: Irgacure 2959 (I-2959) and lithium phenyl-2,4,6-trimethylbenzoylphosphinate (LAP). We chose to compare the two photoinitiators to evaluate their effects on hydrogel properties and to find the most suitable option for our system. I2959 is a classic photoinitiator that is commonly employed in tissue engineering because of its low cytotoxicity and immunogenicity.^42,43^ LAP is a photoinitiator that highly soluble in water and has high cross-linking efficiency at lower concentrations compared to I2959.^36^ We employed ultraviolet (UV) light to cross-link hydrogels here, but LAP can also be activated by visible light to further enhance cytocompatibility.^44,45^

We aimed to investigate the feasibility of applying photo-cross-linkable chitosan/PVA methacrylate hydrogels as a tissue engineering scaffold platform. To that end, we characterized degradation properties and the ability to encapsulate cells within the hybrid hydrogels. Additionally, the influence of hydrogel variables on mechanical, thermal, structural, and antibacterial properties was characterized, and a scratch wound healing assay was employed to explore the influence of these hydrogels on cell migration. This system could provide a simple and affordable platform for tissue engineering scaffolds with tunable properties and long-term cell viability.

## 2 Methods

### 2.1 Materials

All cell culture supplies and chemicals were purchased from ThermoFisher (Waltham, MA) and used as received, unless otherwise indicated.

### 2.2 Material Modification

Chitosan (85% deacetylated, 1527 g/mol) was dissolved (1.5 wt %) in deionized (DI) water with 1% acetic acid for 3 h. Methacrylic anhydride (4 v/v%) was added dropwise and reacted overnight at 40 °C. The solution was dialyzed for 3 days using 3.5 kDa MWCO dialysis kits and freeze-dried to obtain chitosan methacrylate (ChiMA). ChiMA was stored at room temperature for further use. PVA (25 000 g/mol, Polysciences, Warrington, PA)) was dissolved in dimethyl sulfoxide (DMSO) at 90 °C for 1 h. Then, 2-isocyanatoethyl methacrylate was added dropwise and allowed to react for 4 h. The product was precipitated in cold acetone twice to obtain PVA methacrylate (PVAMA). PVAMA was stored in a freezer until further use.

### 2.3 Spectroscopic Analysis

Fourier transform infrared (FTIR) and ^1^H nuclear magnetic resonance (NMR) spectroscopy were used to confirm the structure of ChiMA and PVAMA. Dried samples were scanned from 4000 to 400 cm^–1^ at a resolution of 4 cm^–1^ using FTIR (iD7 ATR, Thermo Scientific, Waltham, MA). For NMR, samples were dissolved in D_2_O, and spectra were obtained using an Advance III HD 400 MHz NMR (Bruker). The degree of substitution (DS) was calculated for ChiMA based on the methacrylate vinyl proton peak areas, δ = 5.4 (s, H1) and 5.6 (s, H2), relative to the area of the proton peaks associated with the chitosan backbone, δ = 3.5–4.0 (s, H3–H6). For PVAMA, methacrylation was calculated based on ^1^H NMR spectrum using the ratio of the area of the methacrylate vinyl proton peaks, δ = 6.1 (s, H1) and 5.8 (s, H2) to the area of the protons peak associated with the PVA backbone, δ = 4.0 (s, H3).^46^

### 2.4 Photopolymerization

ChiMA and PVAMA solutions were combined in seven different ratios (1:0, 1:2, 1:3, 1:1, 3:1, 2:1, and 0:1) to provide a hydrogel library of 100% ChiMA, 3:1 Chi:PVAMA, 2:1 Chi:PVAMA, 1:1 Chi:PVAMA, 1:2 Chi:PVAMA, 1:3 Chi:PVAMA, and 100% PVAMA, respectively (Figure S1). After initial characterization of swelling and mechanical properties, 100% ChiMA, 1:1 Chi:PVAMA, 1:2 Chi:PVAMA, and 100% PVAMA were utilized in the majority of the characterizations as representative samples that enabled analysis of the effects of component content. I-2959 (0.4 wt %) was added to the polymer solutions and mixed for 30 s at 3500 rpm using a speed mixer (Flacktek, Landrum, SC)) before cross-linking under UV light for 15 min to obtain solid hydrogels. LAP (0.016 wt %) was added to a second set of polymer solutions, which were speed mixed and cross-linked under UV light for 3 min. Due to the higher water solubility and shorter required UV exposure time, LAP was selected for the encapsulation process described in Section 2.12.

### 2.5 Gel Fraction and Swelling Ratio

Cylindrical samples (*n* = 3, ∼8 mm diameter, ∼3 mm height) were air-dried for 24 h after cross-linking. Then, the samples were dried in a vacuum oven (50 °C) for 24 h. Samples were weighed (Wi) and placed into a well plate with DI water on a shaker Table (60 rpm, 37 °C) for 24 h to obtain equilibrium swelling and remove any unreacted portions. The water was removed, and the hydrated samples were weighed (Ww). Then, samples were air-dried for 24 h and vacuum-dried at 50 °C for another 24 h. The samples were then reweighed (Wf). Swelling ratio and gel fraction were calculated using the equations:
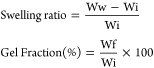


### 2.6 Mechanical Properties

Cylindrical samples (*n* = 3) with height ranging from 4 to 7 mm and diameter of 8–10 mm were fully swollen in 37 °C DI water for 24 h in well plates prior to compressive testing (Model 100P Universal Testing Machine, Test Resources, Shakopee, MN). The machine was set up with a jog rate of 500 mm/min, a preload rate of 5 mm/min, and a control rate of 1 mm/min. Then, the Young’s modulus was calculated using the resulting stress–strain curves.

### 2.7 Thermal Properties

Differential scanning calorimetry (DSC, Q-200, TA Instruments, Inc., New Castle, DE) was used to assess the glass transition temperature (Tg) of the dried hydrogels. Dried samples (3–5 mg, *n* = 3) were put into t-zero aluminum pans and then equilibrated at −40 °C, heated to 120 °C at 10 °C/min, kept isothermally for 2 min, cooled to −40 °C, kept isothermally for 2 min, and then heated to 120 °C at 10 °C/min. Tg was obtained as the half-height of the endothermic transition that occurred during the second heating cycle by using TA Universal Analysis software.

### 2.8 Microstructures

Scanning electron microscopy (SEM, Jeol NeoScope JCM-5000) was utilized to observe the microstructure of the hydrogel samples. Hydrogels were fully swollen in DI water (37 °C) for 24 h. Then, the samples were frozen at −80 °C for 24 h. The frozen hydrogels were placed in a lyophilizer (FreeZone 2.5 Liter, Labconco Corporation, MO) for 24 h to freeze-dry. Samples were cut into 5 × 5 × 3 mm pieces and coated with gold for 45 s using a high vacuum sputter coater (Denton). Microstructures were observed under 10 kV accelerating voltage at 60× magnification.

### 2.9 Degradation Profiles

The degradation behavior of the hydrogels was investigated in phosphate-buffered saline (PBS) and lysozyme (800 mg/L of PBS). Initial sample weights (Wi) were recorded after samples were fully swollen in PBS at 37 °C overnight, and then, samples were placed in media (*n* = 3). The swollen sample mass was measured every 5 days, and fresh solutions were supplied twice a week. Swollen mass remaining was calculated at each time point (*n*) by using equation:
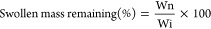


### 2.10 Cytocompatibility

NIH/3T3 fibroblasts were cultured in Dulbecco’s modified Eagle’s medium supplemented with 10% fetal bovine serum and 1% penicillin/streptomycin (Pen/Strep). Cells were seeded into 24 well plates at a density of 5000 cells per well 1 day prior to testing. Dried samples were sterilized by placing them into a UV box for 2 h. Samples were then placed into Transwell inserts above preseeded cells and incubated with the cells for 24 h. A LIVE/DEAD Cell Imaging Kit (488/570) (Thermo Fisher Scientific, Waltham, MA) was used to stain cells. The live cells (green) were determined based on activity enzymatic conversion of calcein AM, while the dead (red) was detected based on cell-impermeant factor bobo-3 iodide. An inverted microscope (Leica DMI6000B, Leica Microsystems Inc., Deerfield, IL) was utilized to take images. Fluorescent micrographs of resulting green and red cells were used to quantify viability of cells by using ImageJ to count cells in each image.

### 2.11 Scratch Wound Healing Assay

NIH/3T3 GFP fibroblasts were seeded into 96 well plates at 5000 cells/cm^2^ and cultured for 3 days in DMEM with 10% FBS and 1% Pen/Strep. Once they reached 90–100% confluency, cell media was removed, and a 10 μL pipet tip was used to scratch a line in the middle of each well from top to bottom. Debris and leftover cells were washed off by using cell media. Hydrogel samples (I-2959 only) were cut into 6 mm diameter cylinders (1 mm thick). Samples were sterilized by UV light for 2 h before swelling in sterile PBS. Then, hydrogel samples were placed on top of the damaged cell layer directly after washing. Media was added back to the wells, and an inverted microscope (5× magnification) was utilized to take images at 1, 24, 48, and 72 h of contact time. ImageJ was used to calculate the cell coverage of the wound area over time.

### 2.12 Cell Encapsulation

NIH/3T3 fibroblasts were used for cell encapsulation experiments.^47^ Briefly, 400 mg of ChiMA was dissolved in 4.950 mL of Dulbecco’s phosphate-buffered saline (DPBS) with 50 μL of acetic acid for 24 h at room temperature. In parallel, 400 mg of PVAMA was dissolved in DMEM for 1 h at 90 °C. Both polymer solutions were mixed for 60 s at 3500 rpm. Approximately, 100 μL of ammonium hydroxide was used to neutralize the pH to 7, and then, 100 μL of LAP solution (8 mg/mL DPBS) was added, and the hydrogel solution was mixed again for 60 s at 3500 rpm. NIH/3T3 fibroblasts (1 × 10^6^ cells/mL) were suspended in 965 μL of DPBS and then gently mixed into the solution. The combination was then placed into 48 well plates (150 μL/well) and cross-linked under UV light for 3 min to obtain solid hydrogels. Collagen hydrogels were prepared as a positive control according to manufacturer guidelines. Briefly, 400 μL collagen (TeloCol-3, Advanced BioMatrix) (3 mg/mL) was combined with 10× DMEM. Subsequently, approximately 8–10 μL of neutralization solution (from the manufacturer) was added to neutralize the pH. pH strips were used to guide adjustment of the pH of the solution to ∼7.4. NIH/3T3 fibroblasts (1 × 10^6^ cells/mL) were suspended in 50 μL of media and gently mixed into the collagen solution. The solution was incubated for 30 min at 37 °C to obtain collagen hydrogels (final collagen concentration: 1.8 mg/mL). Viability of encapsulated cells was assessed at 1, 3, 8, and 14 days. Hydrogel samples were cut to 1 mm thickness then stained with a LIVE/DEAD Cell Imaging Kit (488/570) for 30–45 min. Samples were washed with PBS three times before imaging, as described in Section 2.10.

The morphology of encapsulated NIH/3T3 cells was observed using SEM. Samples were fixed in 2.5% glutaraldehyde (4 °C) for 48 h. Samples were then washed twice with PBS. Then, samples were washed with 30, 50, 70, 90, and 100% ethanol in water (30 min/step) to dehydrate. Samples were dried under vacuum at room temperature for 24 h and coated with gold for 45 s using a high vacuum sputter coater (Denton) before observed via SEM under 15 kV accelerating voltage at 200×, 1000×, and 2700× magnification.

### 2.13 Antimicrobial Properties

Hydrogels were fabricated directly in the wells of 96 well plates (100 μL of solution/well). Samples were then washed in PBS at 37 °C for 24 h to wash out any unreacted byproducts. Samples were air-dried overnight and placed into vacuum oven for 24 h before being sterilized by UV light exposure for 1 h before starting the antimicrobial experiment.^48^*Staphylococcus aureus* (*S. aureus*, ATCC 51153) was cultured overnight in 10 mL Luria–Bertani (LB) broth at 37 °C. Then, 1 mL was taken and cultured in fresh LB until the bacteria reached an optical density at 600 nm (O.D._600_) of 0.6. Bacteria solutions (100 μL) were added to sterilized hydrogels in a well plate at 37 °C for 1 h. Fresh LB was used to diluted bacterial solution by 10^8^. Afterward, the diluted solution (10 μL) was dropped onto a LB-agar plate and then cultured at 37 °C for 18 h. Images were taken, and resulting colony forming units (CFUs) were counted using ImageJ. Bacteria solutions without samples and with QuickClot Combat Gauze were utilized as negative (nonantimicrobial) controls, and bacteria incubated with a clinical wound dressing that contains silver nanoparticles were used as a positive (antimicrobial) control.

### 2.14 Statistics

All experiments were repeated three times. One-way ANOVA followed by student’s *t* test between groups was performed. Significance was set at *p* < 0.05. Results are reported as mean ± standard deviation.

## 3 Results and Discussion

### 3.1 Synthesis

**Figure 1:**
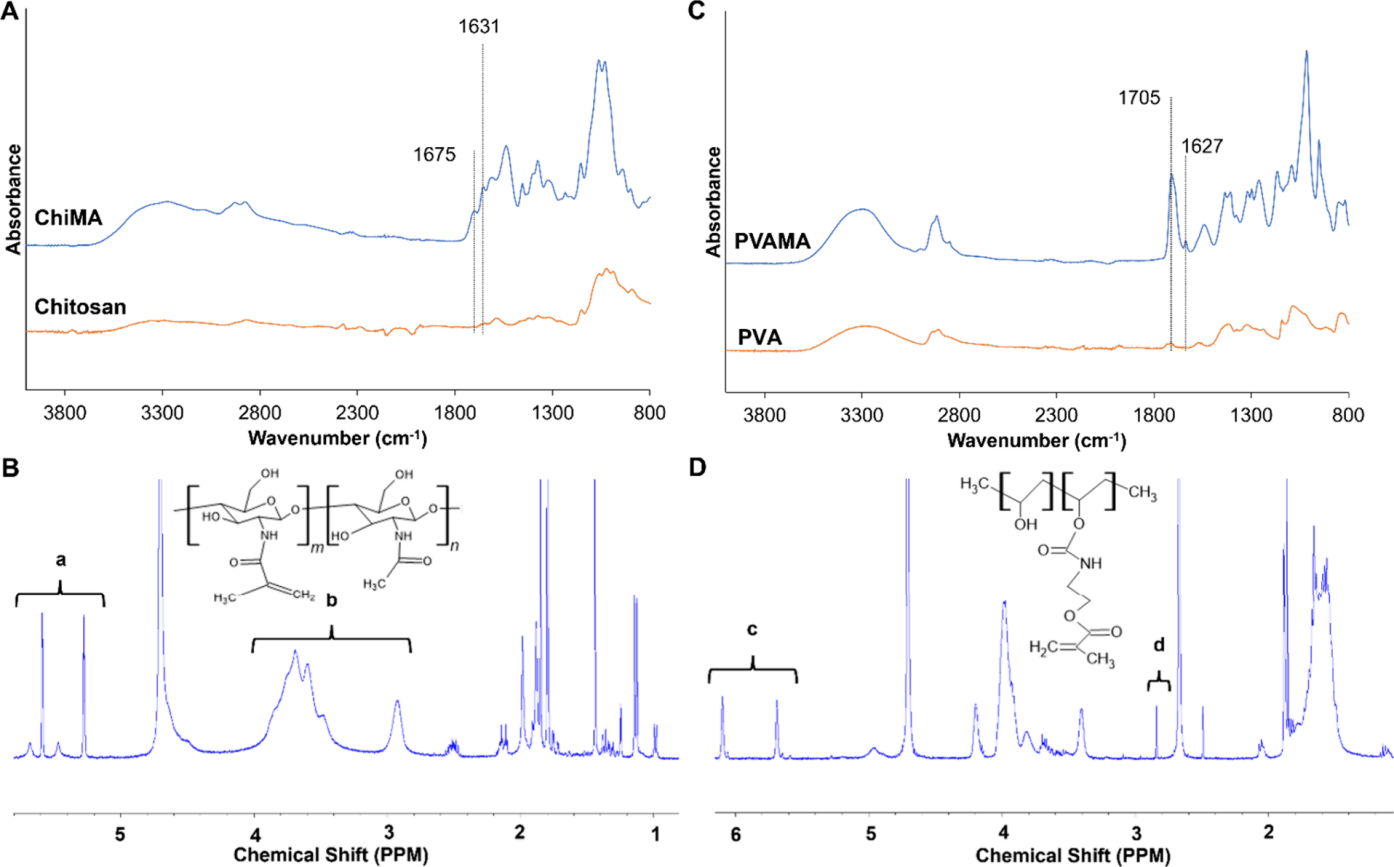
Chemical analysis of ChiMA and PVAMA. (a) Fourier transform infrared (FTIR) and (b) ^1^H-nuclear magnetic resonance (NMR) spectra of ChiMA. (c) FTIR and (d) NMR spectra of PVAMA.

The FTIR spectra of ChiMA in Figure 1A show a peak at ∼1675 cm^–1^, which corresponds with the alkenyl C=C group, and a peak at 1631 cm^–1^, which represents the amide C=O stretch. The addition of these two peaks indicates that ChiMA was successfully obtained. In NMR analysis of ChiMA, the vinyl groups of methacrylate were detected around 5.2–5.8 ppm, and the glucosamine ring was verified by peaks at 2.8–3.8 ppm. The degree of substitution of chitosan hydroxyls (OH) with methacrylate groups was approximately 39% based on H^1^–NMR (Figure 1B), which is similar to previous reports.^49^Figure 1C demonstrates peaks at 1705 and 1627 cm^–1^, illustrating the C=O bond and C=C stretch, respectively, to indicate that PVAMA was synthesized. Successful modification of PVAMA was further confirmed by vinyl peaks at 5.6 and 6.2 ppm and an amine peak at 2.8 ppm in the NMR spectra. For PVAMA, the degree of OH substitution was calculated as approximately 3% (Figure 1D).^47^

### 3.2 Hydrogel Characterization

**Figure 2:**
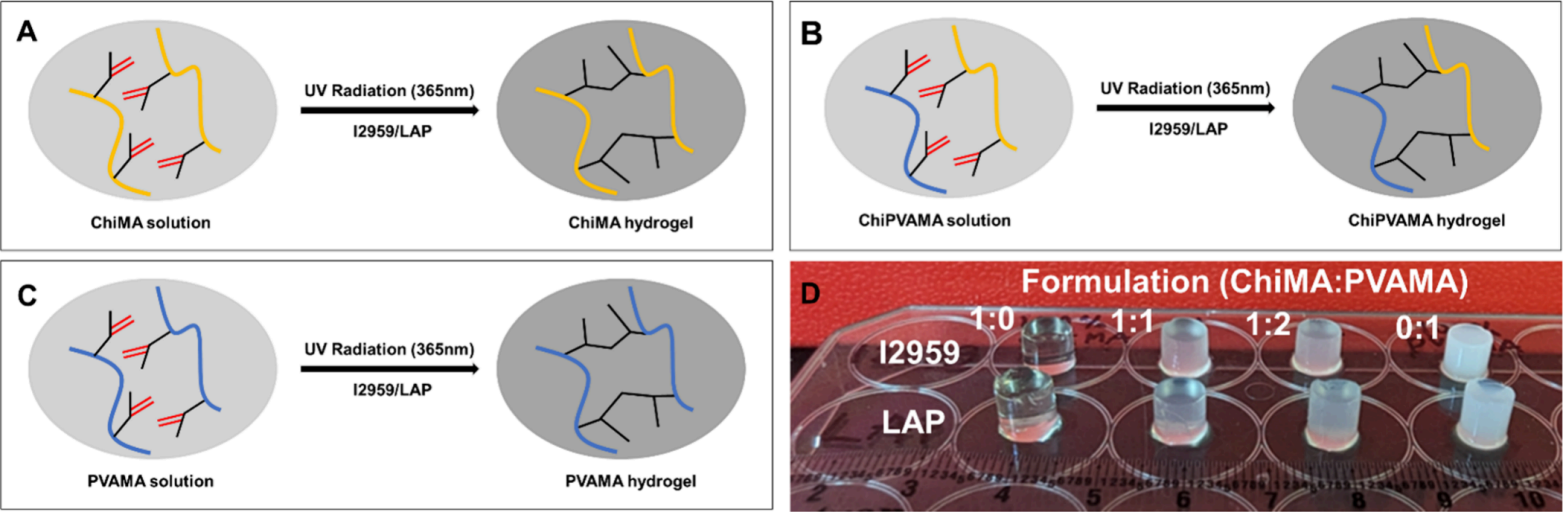
ChiPVAMA hydrogel fabrication. Schematic representation of (a) ChiMA hydrogels from ChiMA solution, (b) ChiPVAMA hydrogels from ChiMA/PVAMA solution, and (c) PVAMA hydrogels from PVAMA solution. (d) Images of hydrogels synthesized with I2959 (back row) and LAP (front row) after 24 h in PBS.

Figure S1 presents the library of ChiPVAMA hydrogel systems right after curing. Figure 2 provides an overview of hydrogel fabrication with images that demonstrate that the synthesized hydrogels preserve their shape after 24 h of swelling in PBS. ChiPVAMA hydrogels can be constructed into intended shapes by using design molds and/or by cutting samples after fabrication, which is a beneficial feature for use in tissue engineering scaffolds.^50,51^ In general, gel fractions of I-2959 hydrogels were around 70%, while gel fractions of LAP hydrogels were higher at ∼80–90%, Table 1. Similar gel fraction ratios were observed in previous studies,^52^ demonstrating successful cross-linking of both ChiMA and PVAMA by I2959 and LAP. The similarity of gel fractions within each initiator group shows that all combinations of natural (ChiMA) and synthetic (PVAMA) polymers provided homogeneous solutions for fabricating consistent hydrogels.

**Table 1:** ChiPVAMA Hydrogel Swelling and Mechanical and Thermal Properties^a^

	Formulation							
	100% ChiMA		1:1 Chi:PVAMA		1:2 Chi:PVAMA		100% PVAMA	
Initiator	I-2959	LAP	I-2959	LAP	I-2959	LAP	I-2959	LAP
Gel Fraction (%)	70 ± 7	93 ± 1	71 ± 2	83 ± 1	73 ± 3	83 ± 1	69 ± 2	84 ± 2
Swelling Ratio	7.2 ± 1.0	5.2 ± 0.9	6.8 ± 0.8	6.2 ± 1.3	6.4 ± 1.2	5.2 ± 0.9^†^	5.8 ± 0.9*	3.3 ± 0.3
Young’s Modulus (kPa)	45 ± 4*	31 ± 2^†‡^	17 ± 2*^†‡^	11 ± 3^†‡^	24 ± 8*^†^	16 ± 4^†‡^	6 ± 1*^†^	3 ± 1^†‡^
Tg (°C)	–	–	57 ± 15	70 ± 3^†^	73 ± 7^†^	70 ± 7^†^	52 ± 4	50 ± 3

a *n* = 3, mean ± standard deviation displayed. *p < 0.05 relative to corollary LAP formulation. ^†^*p* < 0.05 relative to corollary 100% ChiMA sample within initiator group. ^‡^*p* < 0.05 relative to corollary 100% PVAMA sample within initiator group.

All samples showed high water absorption capabilities, with swelling ratios ranging from 7.2 ± 1.0 (I-2959; 100% ChiMA) to 3.3 ± 0.3 (LAP; 100% PVAMA). There is no significant difference between swelling ratios at 24 and 48 h, indicating that equilibrium swelling is achieved by 24 h, Figure S2. The swelling ratio of samples synthesized using I-2959 as the photoinitiator was higher than those of corollary LAP samples, Table 1, which correlates with lower gel fractions that result in more free space in the hydrogel networks. This result tracks with previous work by Khan et al. that demonstrated that higher cross-link density decreased the swelling ratio of chitosan/PVA hydrogels.^53^ Additionally, swelling ratios increased with increasing ChiMA content, which is attributed to the hydrophilic functional groups in ChiMA.^54^ Hydrogels have been extensively scrutinized as extracellular matrix (ECM) mimics based on high water content in these scaffolds.^55^ Based on their high swelling ratios, our ChiPVAMA hydrogel system could be suitable for use as artificial ECM in future work.^56^

Mimicking 3D networks for cellular matrices has remained a challenge in tissue engineering due to the complexity of mechanical properties of native tissue.^57,58^ In tissue engineering, living cells can adhere to the surrounding scaffold through a range of incorporated adhesive cues, interact with the matrix, and sense the resistance to deformation by the surrounding environment. Thus, the stiffness of hydrogel-based scaffolds can influence cell behavior.^59^ Here, samples synthesized with I-2959 had significantly higher Young’s modulus than samples synthesized with LAP. For both I2959 and LAP samples, the Young’s modulus of hydrogels with higher portions of ChiMA (e.g., 100% ChiMA, 2:1 Chi:PVAMA) were significantly higher than hydrogels with lower portions of ChiMA (e.g., 1:2 Chi:PVAMA, 100% PVAMA). The Young’s modulus of 100% ChiMA hydrogels were 45 ± 4 and 31 ± 2 kPa for the I-2959 and LAP samples, respectively. The 100% PVAMA samples provided the lowest Young’s modulus of 6 ± 1 and 3 ± 1 kPa for I-2959 and LAP samples, respectively. The higher stiffness of chitosan is attributed to higher −OH functionalization (per NMR analysis) to increase cross-link density in addition to the relatively stiff ring structure and high number of hydrogen bonding sites within chitosan. It is important to note that the hydrogel swelling ratio and stiffness are typically inversely related, and it is therefore difficult to obtain hydrogels with both high swelling and stiffness. The ChiMA system provides a mechanism for increasing both properties due to the unique structure of chitosan.

Overall, ChiPVAMA hydrogels provide a wide range of Young’s modulus values, which could be tailored to match various kinds of native tissue. For example, the elastic modulus of adipose tissue ranges from 1.6 to 5.5 kPa at the macroscale,^60−62^ while the elastic modulus of thyroid tissue ranges from 9 to 50 kPa.^63−65^ By changing the ratio of ChiMA to PVAMA along with altering the photoinitiator type, our hydrogel system provides a simple method to control the hydrogel modulus for tissue engineering applications. In future work, more fine-tuning of stiffness could be done by altering polymer concentrations in water during hydrogel fabrication or changing the molecular weight of PVA.

Thermal properties provide additional information about hydrogel properties, as polymers below their glass transition temperature (Tg) have vastly different stiffnesses than polymers above their Tg. Thermal properties are also important considerations for the storage of hydrogel-based scaffolds. Interestingly, the 1:2 Chi:PVAMA samples had the highest Tg, while the 100% PVAMA samples had the lowest Tg. We hypothesize that the blend of the two polymers resulted in new intrachain interactions that stiffened the polymer chains and increase Tg. Tg values were generally comparable between the two photoinitiator systems, with the exception of a lower Tg for 1:1 Chi:PVAMA synthesized using I-2959. We did not observe any evidence of phase transition on either of the 100% ChiMA samples, as it is likely above the maximum temperature used for testing and therefore do not report a Tg here. It is important to note that these measurements were made using dry samples and that both polymers are plasticized by water to experience drastic reductions in Tg when wet, so these reported Tg values are most relevant to storage and delivery considerations for hydrogels in the dry state.^66,67^

### 3.3 Hydrogel Microstructures

**Figure 3:**
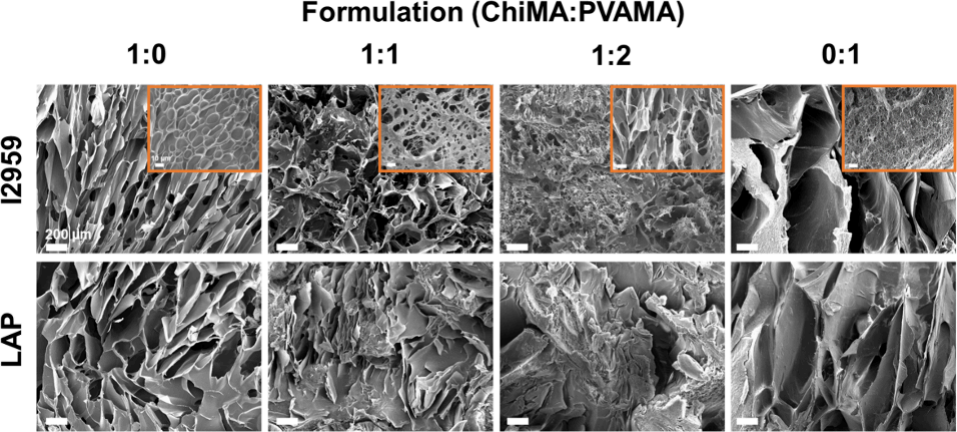
Scanning electron micrographs showing the morphology of lyophilized hydrogels with varied ChiMA:PVAMA ratios and photoinitiator types. Inset SEM images display I2959 hydrogels at 1000× magnification. Scale bars apply to all images.

All hydrogel formulations showed porous structures when visualized by using SEM (Figure 3), with minimal differences observed between the two photoinitiator systems. Qualitatively, 100% PVAMA hydrogels had the largest pores. The samples with only ChiMA or PVAMA produced more uniform structures than blended samples. This result is potentially due to the significant difference between the molecular weights of chitosan and PVA. Under increased magnification, interconnected structures were detected in the blended samples synthesized using I-2959, while the pure chitosan polymer samples had more closed structures (Figure 3). This effect may be due increased cross-linking efficiency between polymer chains in homogeneous solutions, as described previously.^68,69^ The morphology of chitosan and PVA hydrogels was previously investigated, in which pore size ranged from 100 to 1000 μm.^70−72^ Our images confirm that the current ChiPVAMA hydrogel system could be utilized for tissue engineering scaffolds based on lyophilized pore sizes that are larger than 100 μm.^73,74^ It should be noted that these microstructures would be altered when hydrogels are in the hydrated, swollen state. However, it is difficult to obtain high magnification images of the hydrated biomaterials.

### 3.4 Hydrogel Degradation

**Figure 4:**
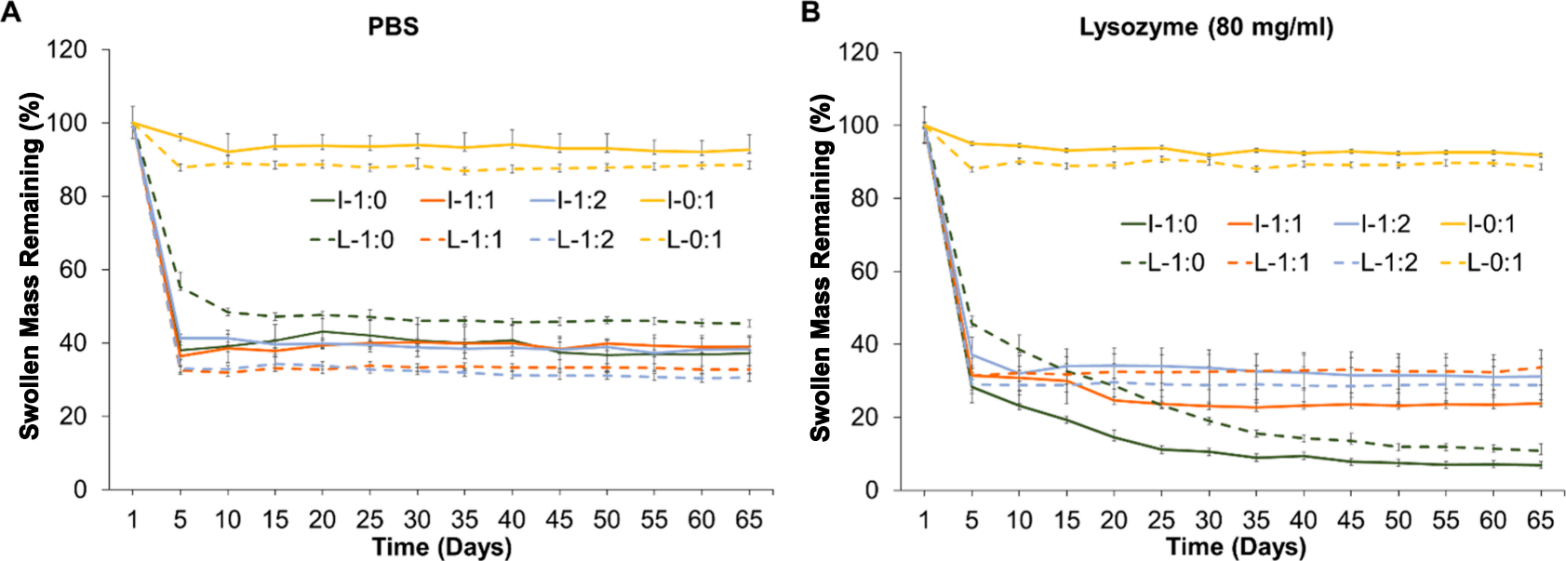
Degradation profiles (swollen mass remaining) of hydrogels. ChiPVAMA hydrogels synthesized with I2959 (I) and LAP (L) after 65 days in (A) PBS and (B) 80 mg/mL of lysozyme (*n* = 3, mean ± standard deviation displayed, ratios in chart legends indicate amount of ChiMA relative to PVAMA).

Figure 4 shows the swollen mass remaining of ChiPVAMA hydrogels in PBS and lysozyme (80 mg/mL) over 65 days of degradation. The 100% PVAMA hydrogels did not degrade in PBS or lysozyme, confirming that the PVA portion of the hydrogels is not hydrolytically or enzymatically degraded. Literature has demonstrated that PVA methacrylate hydrogels can be degraded via hydrolysis of the esters in the methacrylate groups, but this degradation likely occurs over a much longer time frame than we employed in this study.^75,76^ Further studies in accelerated media could be performed to explore this property more fully.

After 65 days in the lysozyme solution, the swollen mass remaining for 100% ChiMA samples synthesized with I2959 and LAP was 7 ± 1 and 11 ± 1%, respectively, Figure 4B. This result confirms that lysozyme can break down the chitosan portions of the hydrogel. Lysozyme hydrolyzes β(1–4) glycosidic bonds, which results in breakdown of chitosan into glucosamine and N-acetyl-glucosamine.^77^ Additionally, chitosan can be hydrolytically degraded in aqueous solutions, including PBS.^78^ Therefore, the observed mass loss of ChiMA-containing hydrogels was expected in both media types, and the accelerated degradation in lysozyme (vs PBS) was anticipated. The heterogeneous and incomplete degradation of chitosan was expected based on previously observations in which the deacetylation degree (DD) of chitosan can affect degradation by lysozyme.^79,80^ In this system, 100% ChiMA hydrogels likely did not completely degrade due to the modifications made to the −OH groups on chitosan during methacrylation.^81^ However, previous studies show that partial DD of chitosan results in highly uniform biodegradability in the body and safe excretion in urine.^82^ Thus, the DD of chitosan could be tuned in future studies to more precisely control the degradation rate. Future studies will also evaluate the degradation byproducts to better understand how these hybrid materials break down over time.

The total observed mass loss is increased with a higher ChiMA content in both types of hydrogels. Furthermore, in I2959 hydrogels, faster degradation was observed in lysozyme as compared with PBS. However, for the blended LAP hydrogels, there was no significant difference in the swollen mass remaining of samples after degradation in PBS and lysozyme solutions. This result may be due to the increased efficiency of LAP as a photoinitiator when compared with I2959, which may result in more homogeneous networks that degrade at more controlled rates. An initial mass drop in the chitosan-containing samples was observed at day 5 for both types of hydrogels, which may be due to early removal of unreacted polymer portions. The degree of methacrylation of chitosan was relatively high, but its bulky structure could negatively impact cross-linking efficiency.^53^ This result generally corresponds with the gel fraction data in (Table 1), in which 10–30% of the polymer content was removed after 1 day of incubation in PBS. However, the observed mass losses were higher than the soluble gel components, demonstrating that degradation occurs in conjunction with potential solubilization of un-cross-linked components. Overall, tunable enzymatic degradation rates could be achieved in this system by altering ChiMA content.

### 3.5 Cytocompatibility and in Vitro Scratch Wound Healing

**Figure 5:**
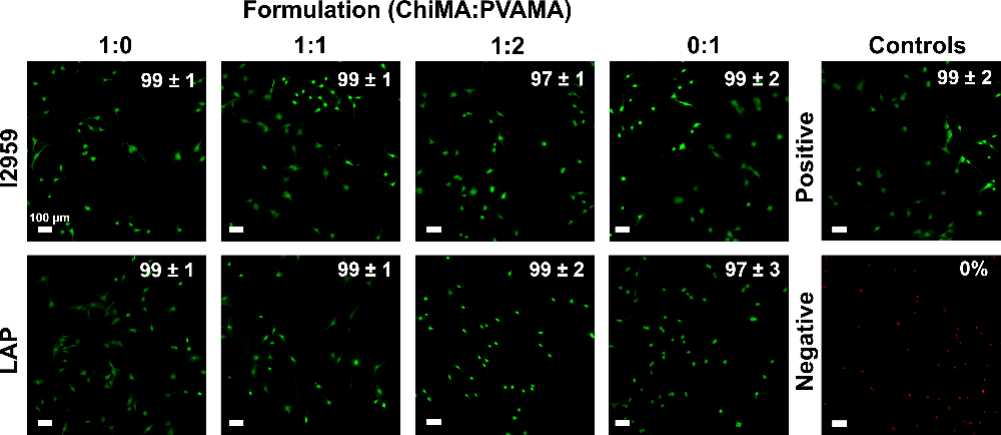
Cytocompatibility of ChiPVAMA hydrogels determined using LIVE/DEAD assay after 24 h of exposure. Scale bar applies to all images. Overlaid numbers are quantified viabilities based on the ratios of live (green) and dead (red) cells. *n* = 3 samples, with 3 images/well for a total of 9 images/formulation. Mean ± standard deviation displayed.

**Figure 6:**
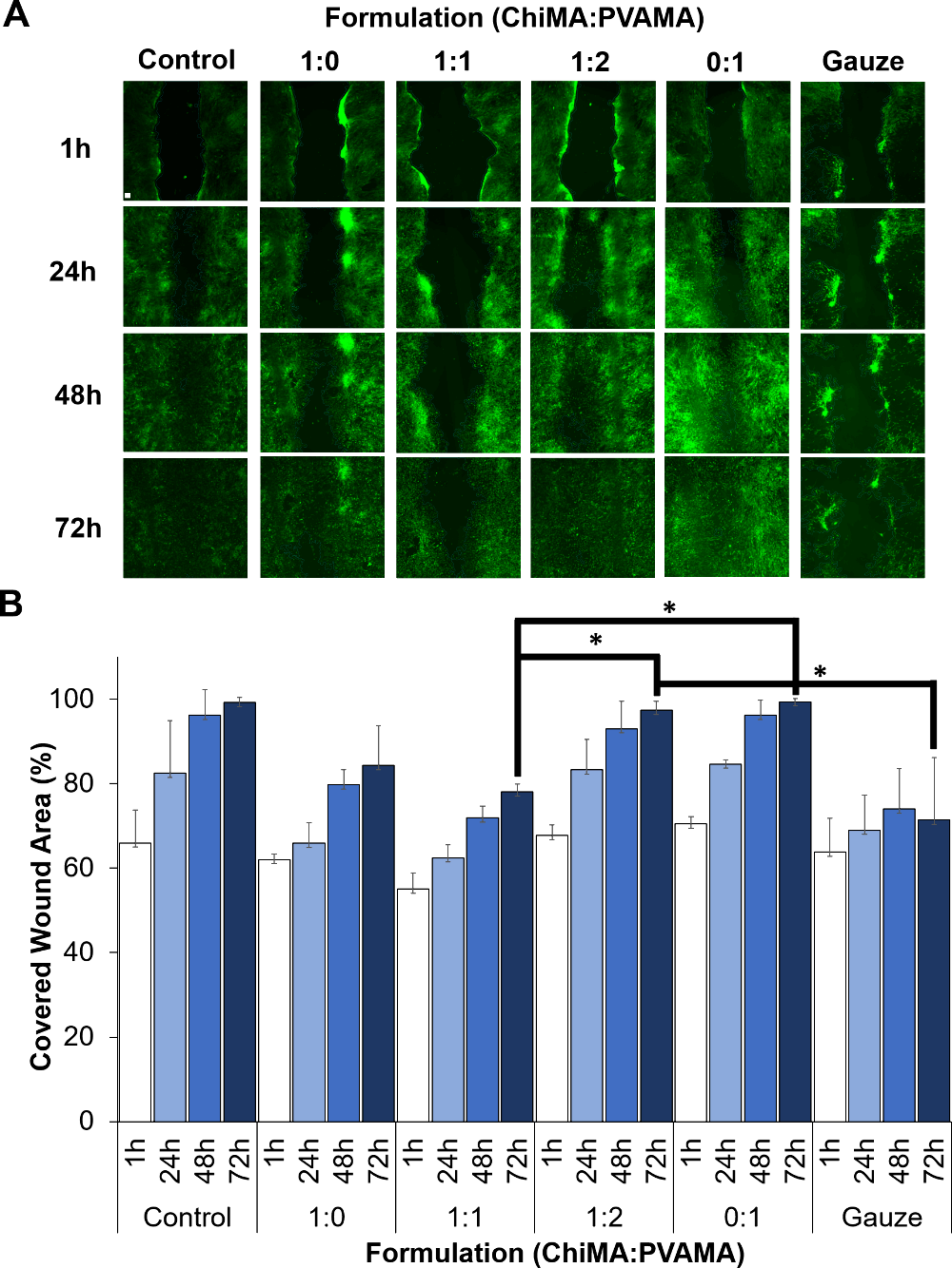
2D in vitro scratch wound healing assay. (A) Microscopic (5×) images of scratch wound healing assay representing the effect of various samples on cell migration (scale bar = 100 μm and applies to all images). (B) Quantified values of covered wound areas at 1, 24, 48, and 72 h (*n* = 3, mean ± standard deviation displayed, *p <0.05).

All hydrogels demonstrate high cytocompatibility (>97%) as compared with the positive control group when characterized using a LIVE/DEAD assay, Figure 5. Once initial cytocompatibility was confirmed, an *in vitro* scratch wound healing assay was carried out to determine the effects of hydrogels on a simple wound healing process, Figure 6A. Two-dimensional wounds made in well plates were exposed to hydrogels, and wound closure was tracked over 72 h. By 48 h, the wounds exposed to 1:2 Chi:PVAMA and 100% PVAMA samples demonstrated cell coverage on wounded surface areas of 93 ± 6 and 96 ± 4%, respectively. However, 100% ChiMA and 1:1 Chi:PVAMA had lower 2D wound coverage at 80 ± 4 and 72 ± 3%, respectively. All hydrogel samples had improved 2D wound closure compared with the gauze clinical control after 72 h (Figure 6B). Overall, hydrogels with more PVAMA content had better effects on cell migration, which may be due to their lower modulus and higher diffusion capabilities. This is a unique feature that could be explored further for wound treatment applications.

### 3.6 Cell Encapsulation

**Figure 7:**
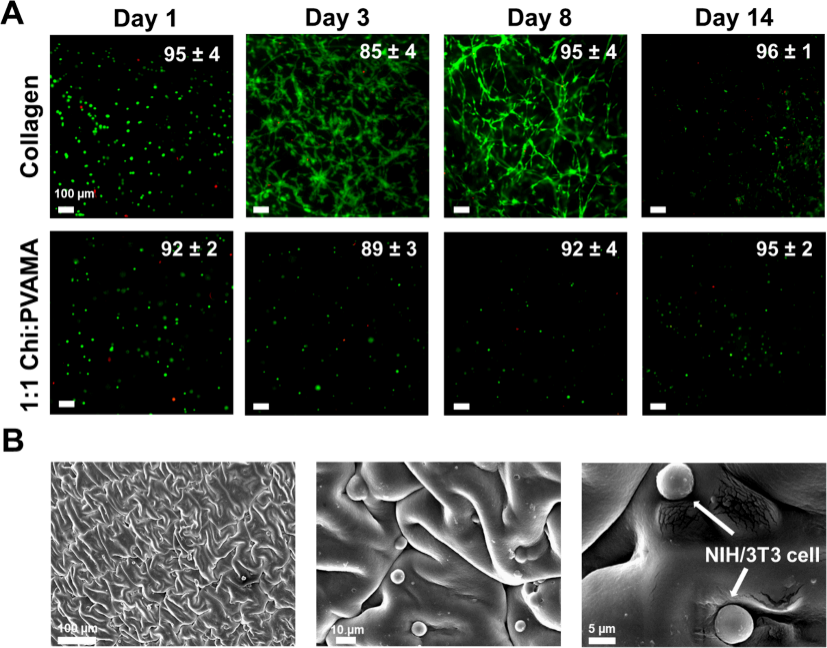
Cell encapsulation within ChiPVAMA hydrogels. (a) LIVE/DEAD assay with NIH/3T3 cells after encapsulation within 1:1 Chi:PVAMA hydrogels as compared with collagen hydrogels over 14 days (*n* = 3, mean ± standard deviation displayed). (b) Scanning electron micrographs of NIH/3T3 cells encapsulated within 1:1 Chi:PVAMA hydrogel (left-200×, middle-1000×, right-2700×).

We encapsulated cells within 1:1 Chi:PVAMA (LAP) hydrogels and characterized their viability as compared with cells encapsulated in collagen control hydrogels, Figure 7. At day 1, the cell viability was similar between collagen and ChiPVAMA hydrogels at 95 ± 4 and 92 ± 2%, respectively. Photoinitiators produce free radicals that can interact with cells during encapsulation and negatively impact viability.^83,84^ The high initial cell viability confirms that the hydrogel solution components, including LAP, are well tolerated by mammalian cells (i.e., 3T3 NIH cells) in this system, which is consistent with other literature observations.^85,86^

There were no significant differences in quantified cell viabilities between collagen and 1:1 Chi:PVAMA hydrogels over the full 14 days of this study, Figure 7A. Cell viabilities were maintained >88% at all time points. However, on days 3 and 8, images of cells within hydrogels show that collagen better supports cell attachment and proliferation, based on larger cell areas and spreading within the collagen gels vs smaller, rounded cells in the ChiPVAMA hydrogels. This result demonstrates that ChiPVAMA hydrogel does not support cell attachment, which is expected due to the lack of cell adhesive sites in chitosan and PVA. On day 14, the number of the cells was reduced in both sample types, which is attributed to collagen degradation during this time frame and the lack of attachment sites for cells in the ChiPVAMA gels. SEM images were employed to observe the morphology of the cells after encapsulation into the hydrogel system, Figure 7B. The NIH/3T3 cells maintain their morphology after encapsulation into the hydrogels and appear to arrange themselves in the hydrogel pores. With the flexibility in the structure of ChiPVAMA polymer, the hydrogel system could be easily modified with biologically active components (e.g., gelatin) in future work to support better cell attachment.

### 3.7 Antimicrobial Properties

**Figure 8:**
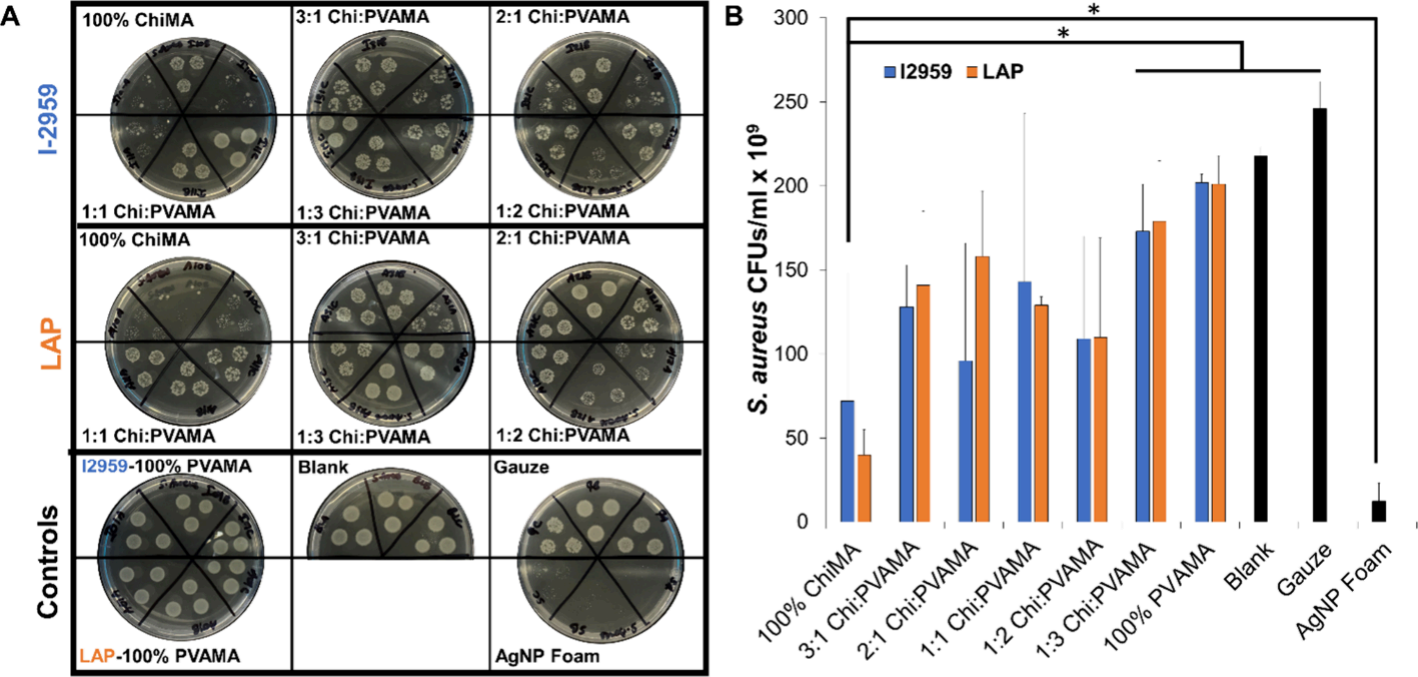
Antimicrobial properties of hydrogels against *S. aureus*. (A) *S. aureus* CFUs after exposure to I2959 (top row) and LAP (middle row) hydrogels as compared with controls (bottom row) after 24 h. (B) Quantified CFUs/mL (*n* = 3, mean ± standard deviation displayed, *p < 0.05).

Chitosan is well-known for being inherently antimicrobial, and infection can negatively impact function of tissue engineering scaffolds.^29,87,88^ Thus, antimicrobial properties of hydrogels were characterized here, as shown in Figure 8. The 100% ChiMA hydrogels with I2959 showed the highest antimicrobial properties as compared with controls and other types of hydrogels when testing with *S. aureus* after 24 h. However, the 100% ChiMA hydrogels were not as efficient at killing bacteria as the clinical control (a wound dressing that contains silver nanoparticles (AgNPs)). In hydrogels with I2959, the decreased ChiMA content correlated with reduced antimicrobial properties. Samples with LAP have better antimicrobial properties than those with gauze and more consistent antimicrobial efficacy among the varied formulations.

Several factors can influence the antibacterial properties of chitosan such as microbial environment, intrinsic properties of chitosan, physical state, and environmental conditions.^87^ Thus, chitosan’s antibacterial capacity is variable. In this case, the 100% ChiMA samples with I2959 had markedly better efficacy. This result might be due to the lower gel fraction of I2959 samples, which promoted increased ChiMA diffusion into the bacteria solution to inhibit the activity of *S. aureus.* A previous study showed that chitosan combined with 4-arm polyethylene glycol in hydrogels has high antibacterial properties against both *Escherichia coli* and *S. aureus*, based on the number of amino groups available in the hydrogel.^89^ These results indicate that ChiPVAMA hydrogels could be further modified (e.g., with antimicrobial components) to enhance their antibacterial properties in future studies.

## 4 Conclusions

The ChiPVAMA hydrogel system displays fascinating and wide-ranging properties for tissue engineering scaffolds, including degradability, tunability, suitability for cell encapsulation, and antimicrobial efficacy. Our study demonstrates that blending ChiMA and PVAMA is a straightforward method to archive a wide range of degradation rates and mechanical properties that can be applied in various tissue scaffold applications. Moreover, the flexibility of I2959 and LAP as photoinitiators also enhanced the tunability of the platform. In addition, our study explored the initial potential for wound healing and antibacterial properties of the ChiPVAMA hydrogel. These properties could be employed to design potential platforms for wound management and implantable tissue scaffolds that aid the healing process in future work.

## Supporting Information Available

Images of full hydrogel library; data for hydrogel swelling at 30 min, 24 h, and 48 h; and swelling and modulus data for full hydrogel library (PDF)
