# Supplementary material for: Chitosan Poly(vinyl alcohol) Methacrylate Hydrogels for Tissue Engineering Scaffolds
Source: ACS Appl Bio Mater. 2024 Feb 21;7(12):7818–27. doi: 10.1021/acsabm.3c01209 (PMC11653253; doi:10.1021/acsabm.3c01209)
Supplement: Supplementary file 1 — mt3c01209_si_001.pdf [file mt3c01209_si_001.pdf]

Supporting Information

Chitosan Polyvinyl Alcohol Methacrylate Hydrogels for Tissue Engineering Scaffolds

Nghia Le Ba Thai[1], Henry T. Beaman[1], Megan Perlman[1], Ernest E. Obeng[1], Changling Du[1], Mary Beth B. Monroe[1]*

[1]Department of Biomedical and Chemical Engineering, Syracuse Biomaterials Institute, and BioInspired Syracuse: Institute for Material and Living Systems, Syracuse University, Syracuse, NY 13244.

*Corresponding author: Dr. Mary Beth Browning Monroe

Department of Biomedical and Chemical Engineering, BioInspired Syracuse: Institute for Materials and Living Systems

Syracuse University

318 Bowne Hall

Syracuse, NY 13244

Tel: (315) 443-3323

E-mail: mbmonroe@syr.edu

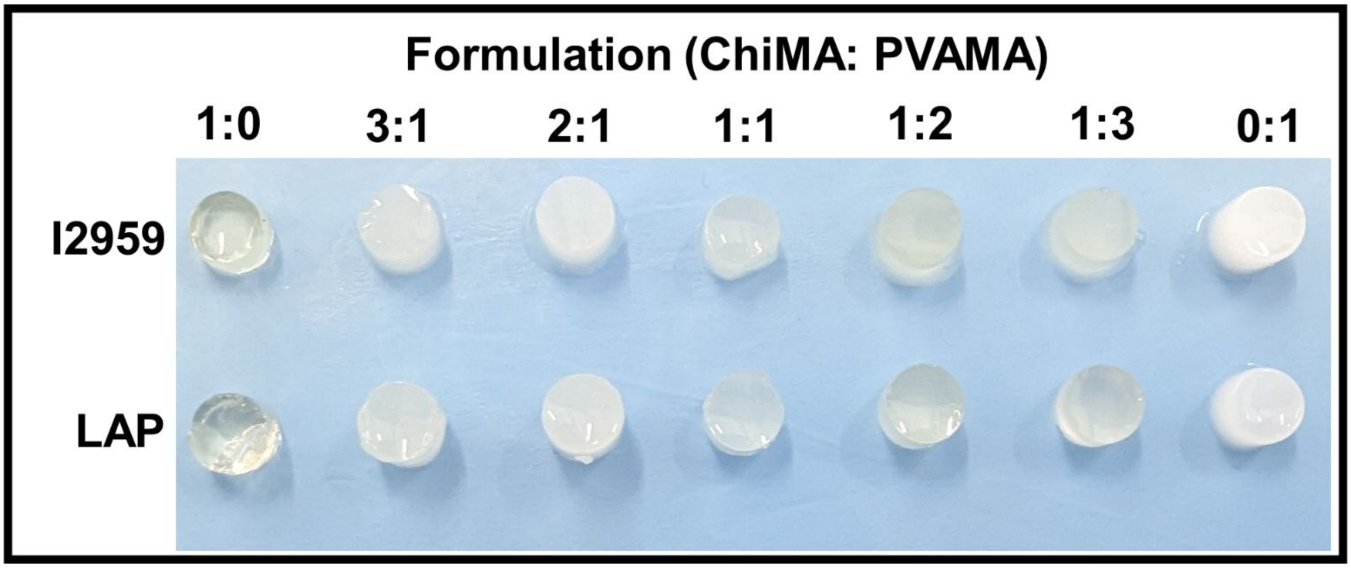

Figure S1. Images of hydrogels synthesized with Irgacure 2959 (I2959, top) and lithium phenyl-2,4,6-trimethylbenzoylphosphinate (LAP, bottom) after curing.

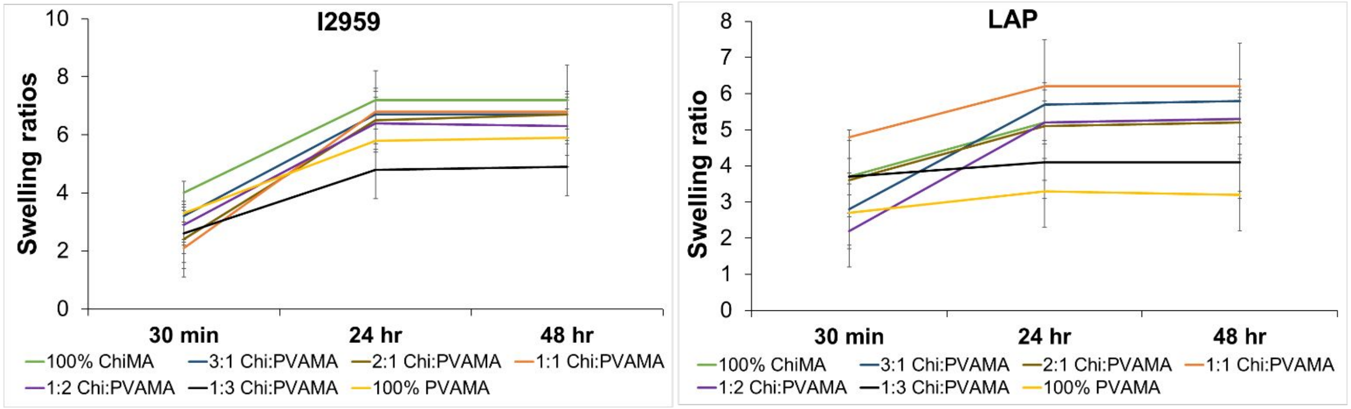

Figure S2. Swelling ratios of ChiPVAMA hydrogels over time (I2959 – left, LAP – right), used to confirm 24 hour time point for equilibrium swelling characterization.

Table S1. ChiPVAMA hydrogel swelling and mechanical properties. n = 3, mean ± standard deviation

displayed.

Formulation (ChiMA: PVAMA)	Initiator	Swelling Ratio	Modulus (kPa)
1:0	I2959	7.2 ± 1.0	45 ± 4
	LAP	5.2 ± 0.9	31 ± 2
3:1	I2959	6.7 ± 0.8	40 ± 6
	LAP	5.7 ± 0.6	17 ± 2
2:1	I2959	6.5 ± 0.8	50 ± 6
	LAP	5.1 ± 0.7	28 ± 5
1:1	I2959	6.8 ± 0.8	17 ± 2
	LAP	6.2 ± 1.3	11 ± 3
1:2	I2959	6.4 ± 1.2	24 ± 8
	LAP	5.2 ± 0.9	16 ± 4
1:3	I2959	4.8 ± 0.9	12 ± 1
	LAP	4.1 ± 0.5	5 ± 1
0:1	I2959	5.8 ± 0.9	6 ± 1
	LAP	3.3 ± 0.3	3 ± 1
